# Correction: MicroRNA22-5p targets ten-eleven translocation and regulates estrogen receptor 2 expression in infertile women with minimal/mild endometriosis during implantation window

**DOI:** 10.1371/journal.pone.0238656

**Published:** 2020-08-28

**Authors:** The PLOS ONE Staff

## Notice of republication

An incorrect version of Fig 4 was published in error. The publisher apologizes for this error. This article was republished on August 10, 2020, to correct for this error. Please download this article again to view the correct version.
