# Supplementary figures and images for: MicroRNA22-5p targets ten-eleven translocation and regulates estrogen receptor 2 expression in infertile women with minimal/mild endometriosis during implantation window
Source: PLoS One. 2020 Jul 13;15(7):e0234086. doi: 10.1371/journal.pone.0234086 (PMC7357761; doi:10.1371/journal.pone.0234086)

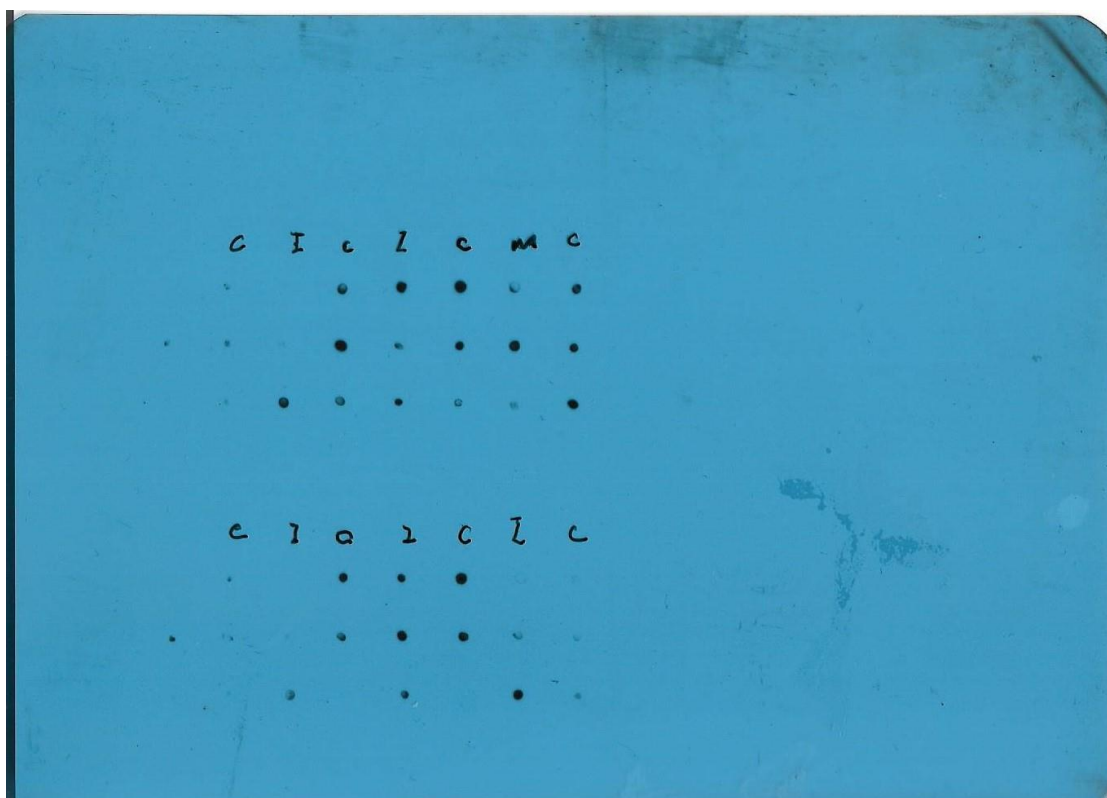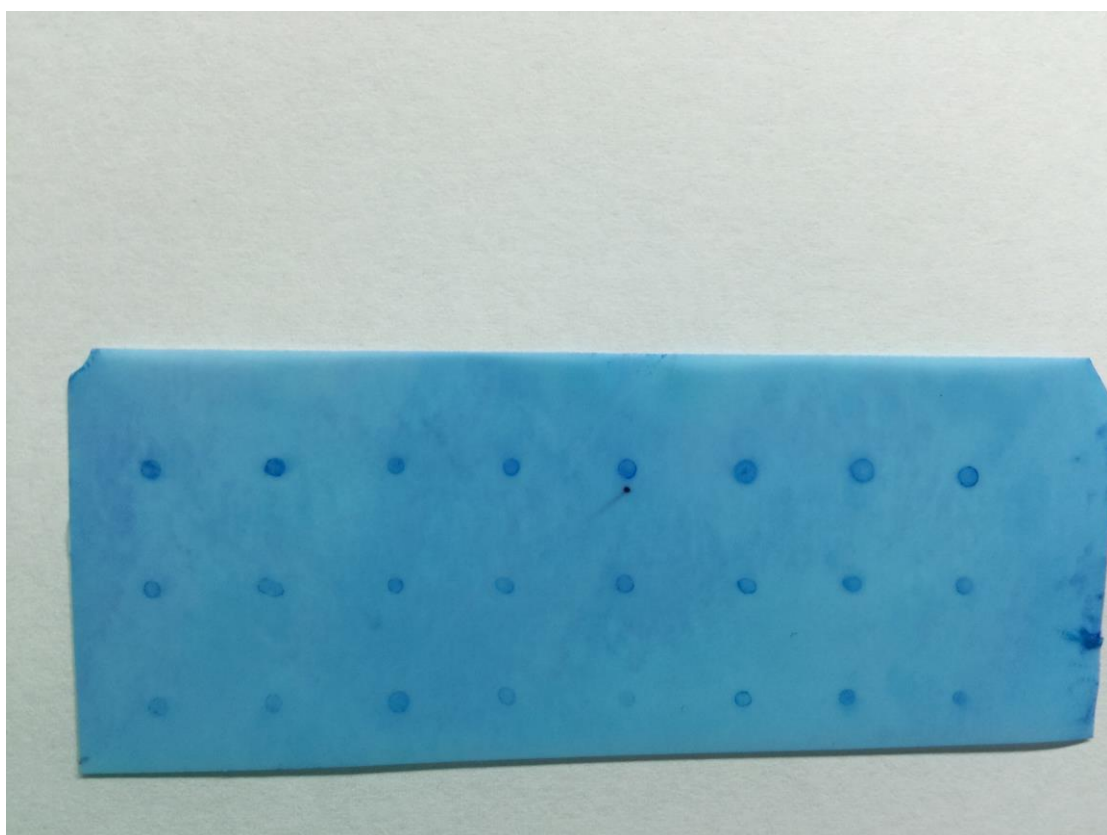

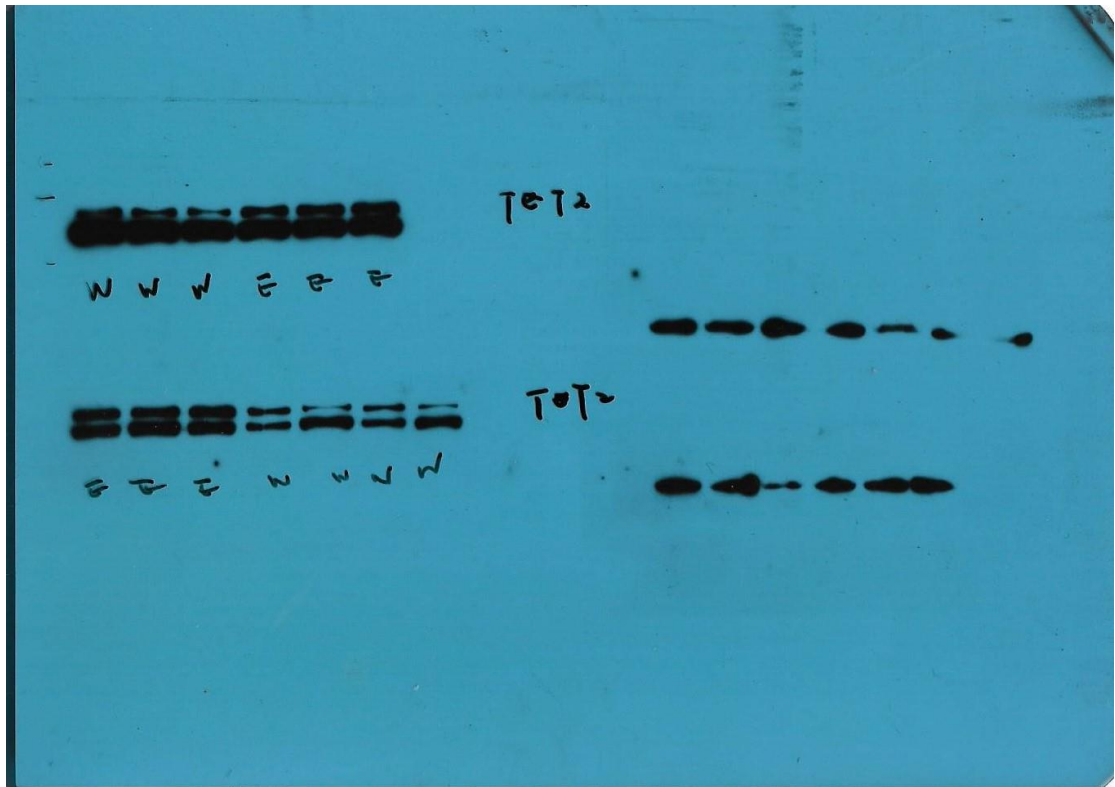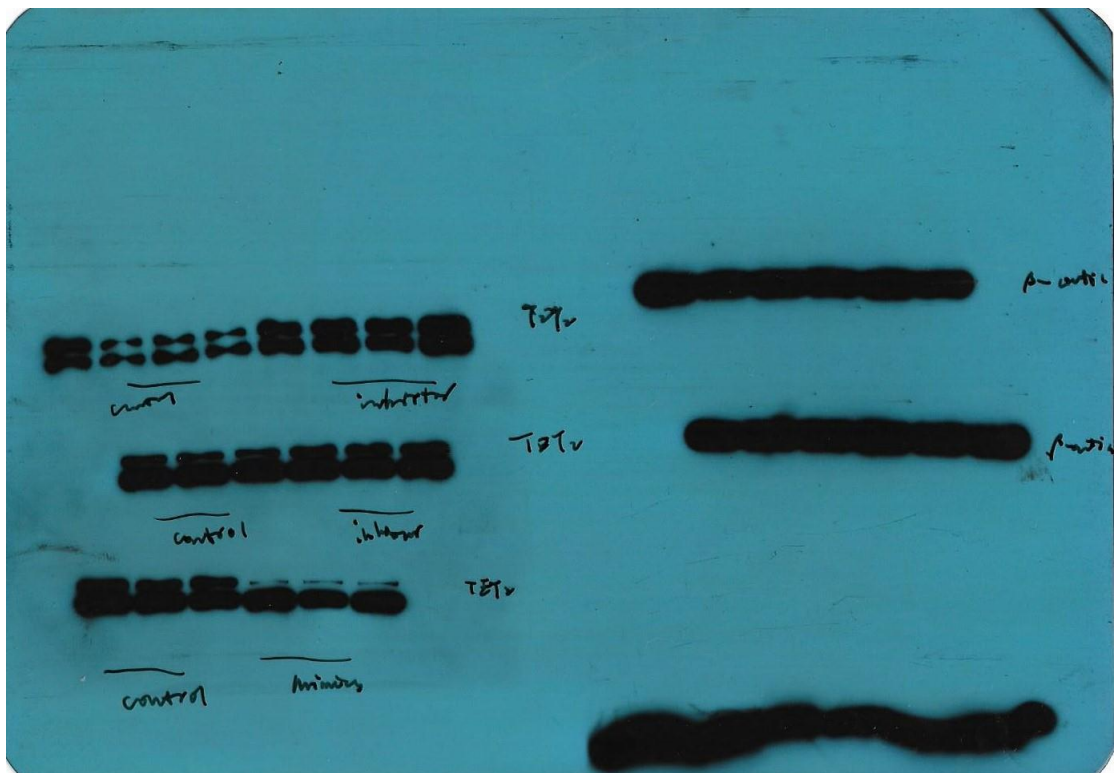

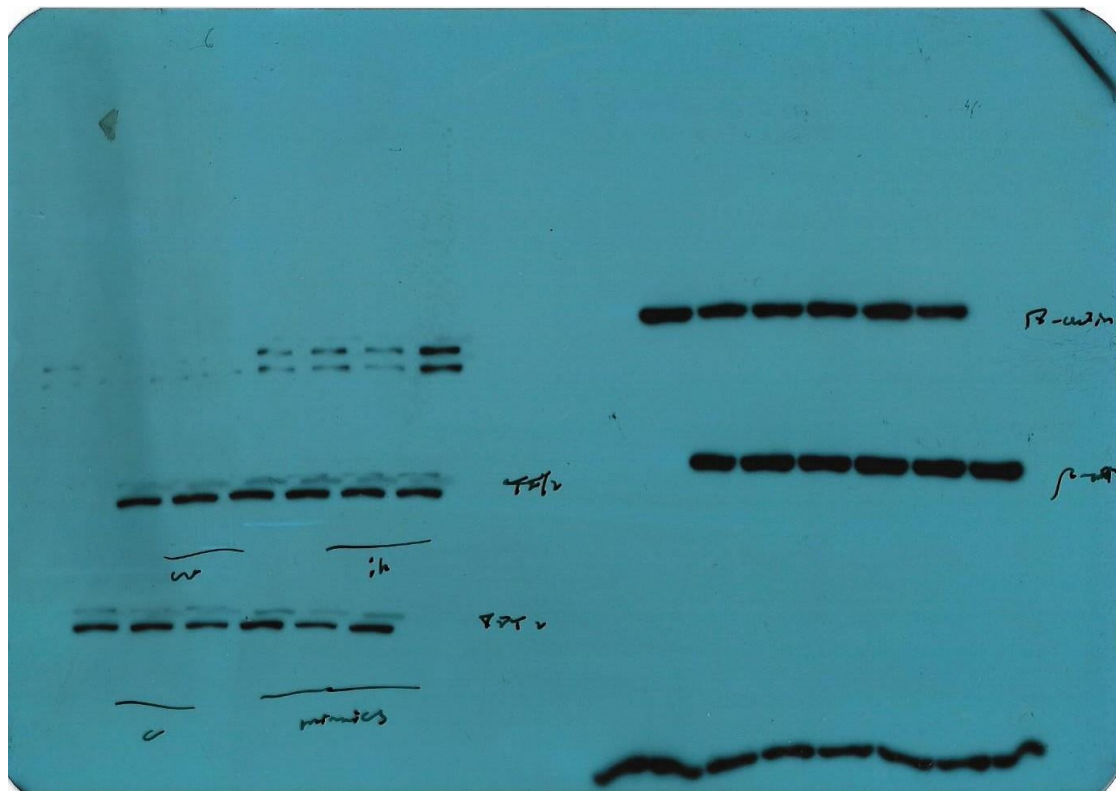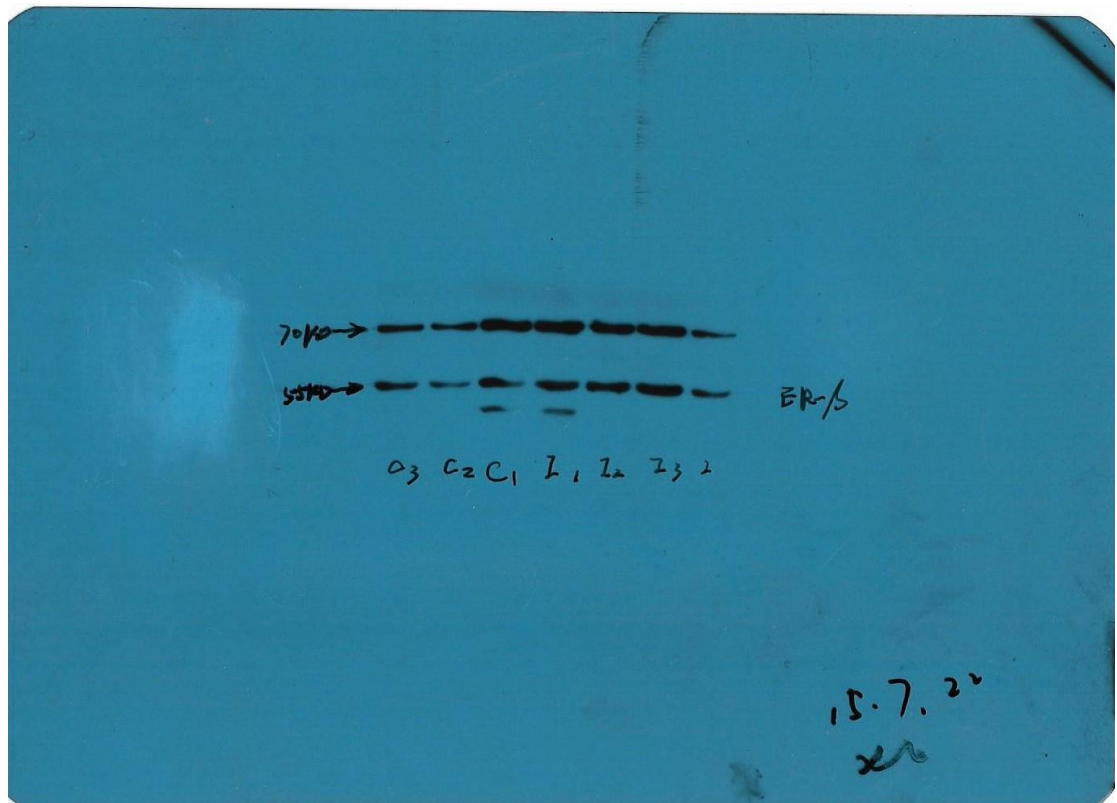

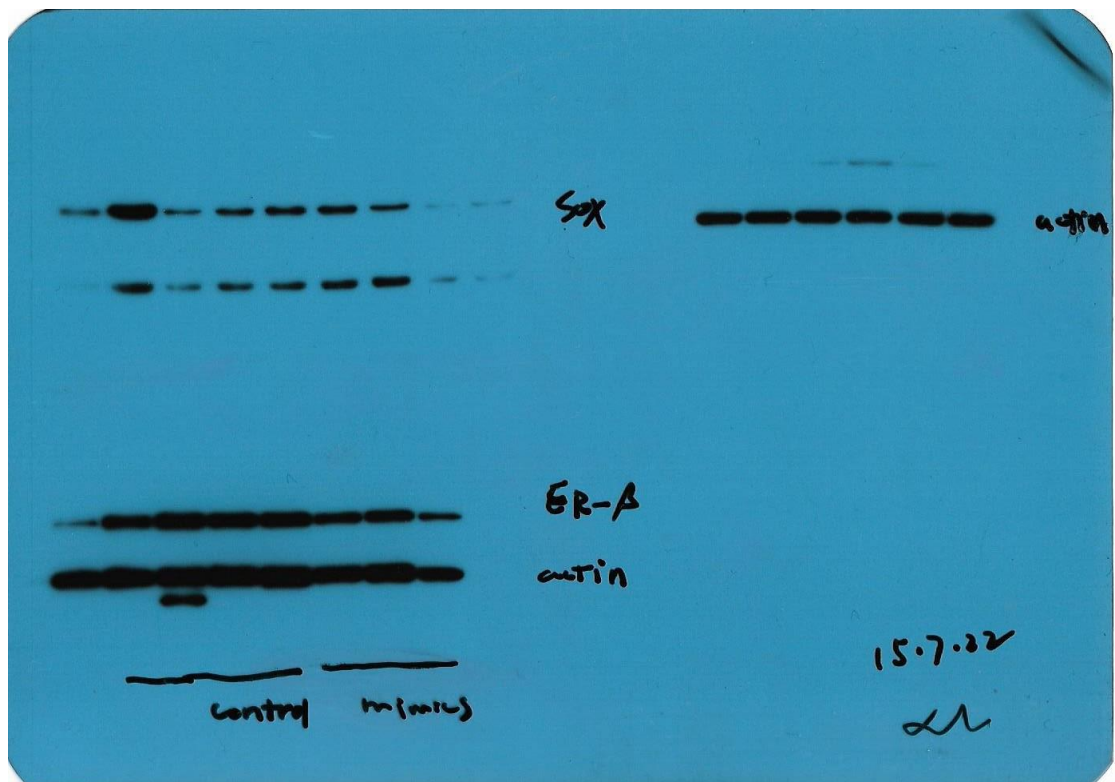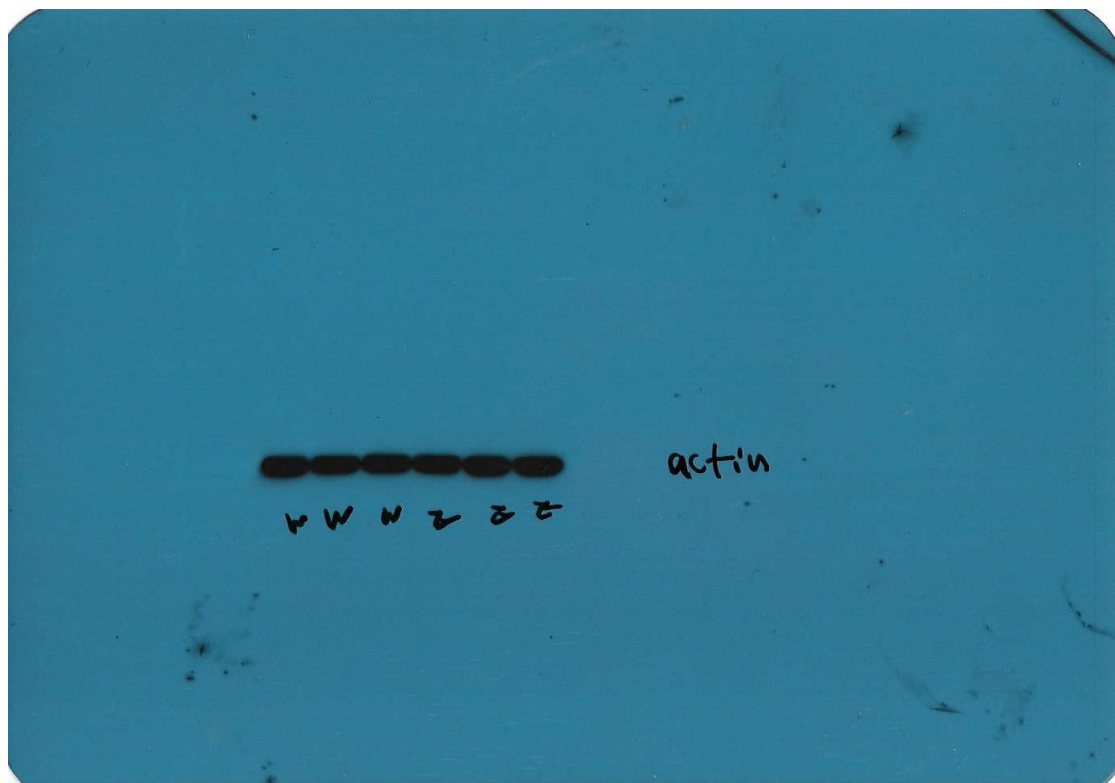

Supplement: S1 Raw images — (PDF) [file pone.0234086.s001.pdf]
