# Supplementary material for: MicroRNA22-5p targets ten-eleven translocation and regulates estrogen receptor 2 expression in infertile women with minimal/mild endometriosis during implantation window
Source: PLoS One. 2020 Jul 13;15(7):e0234086. doi: 10.1371/journal.pone.0234086 (PMC7357761; doi:10.1371/journal.pone.0234086)
Supplement: Attachment — Submitted filename: Responses to reviewers.docx [file pone.0234086.s003.docx]

**Responses to reviewers**

Reviewer #2: The authors are commended for their efforts to address the reviewer’s comments. However, there are some concerns remain.

Major concern # 1: Since the authors know nothing about the function of miR22-5p, it is suggested that this limitation be discussed in the “discussion section”.

**Reply:** The limitation of this study has been discussed in the “discussion section”, which is showing on Page 21, Line 3-11.

Major concern # 6: The language has been improved, but there are still many errors and awkward sentences that prevent a clear understanding of the manuscript. Language editing is strongly recommended. A few examples are listed below:

Page 2, Line 15-16: MiR22-5p regulated the expression of ESR2, but do not directly affect the methylation of ESR2 promoter region. In this sentence, “do not” should be “did not”.
**Reply:** Thanks for figuring out this inappropriate grammar. “do not” has been replaced with “did not”, which is showing on Page2, Lines 15-16.

Page 2, Lines 17-18: This study provides a novel approach for the imbalance of miR22-5p expression in mid-luteal endometrium of minimal/mild endometriosis, may involve in the mechanism of its associated infertility. This sentence is grammatically incorrect.
**Reply:** Thanks for this concern. This sentence has been reorganized in the revised manuscript, and it is showing on Page 2, Lines 17-18.

Page 21, Lines 20-21: Our findings may provide a new novel approach to target in the mechanisms of minimal/mild endometriosis associated infertility. In this sentence, new and novel are repetitive. The sentence is also grammatically inappropriate.

**Reply:** Thanks for this reminding, “new” has been removed in the revision, which could be found on Page 21, Lines 20-21.

**Thanks for the comments on the language. The revised manuscript has been reedited by Editage services (**[**http://app.editage.com/**](http://app.editage.com/)**). We are hoping that English writing in this revision has been improved and meet the required standards for publication.**

While revising your submission, please upload your figure files to the Preflight Analysis and Conversion Engine (PACE) digital diagnostic tool, <https://pacev2.apexcovantage.com/>. PACE helps ensure that figures meet PLOS requirements. To use PACE, you must first register as a user. Registration is free. Then, login and navigate to the UPLOAD tab, where you will find detailed instructions on how to use the tool. If you encounter any issues or have any questions when using PACE, please email us at [figures@plos.org](mailto:figures@plos.org). Please note that Supporting Information files do not need this step.

**Reply:** All figure files have been uploaded the Preflight Analysis and Conversion Engine (PACE) digital diagnostic tool.
